# Nest Site Selection by Green Sea Turtles ( *Chelonia mydas* ) and Implications for Conservation on Qilianyu, Xisha Islands, South China Sea

**DOI:** 10.1002/ece3.70841

**Published:** 2025-01-20

**Authors:** Ting Zhang, Xiaoyu An, Chenglong Zhang, Yunteng Liu, Yupei Li, Yangfei Yu, Jichao Wang, Liu Lin, Hai‐Tao Shi

**Affiliations:** ^1^ Ministry of Education Key Laboratory for Ecology of Tropical Islands, Key Laboratory of Tropical Animal and Plant Ecology of Hainan Province, College of Life Sciences Hainan Normal University Haikou China; ^2^ Hainan Sansha Provincial Observation and Research Station of Sea Turtle Ecology Sansha China; ^3^ Marine Protected Area Administration of Sansha City Sansha China

**Keywords:** green sea turtle, hatching success, microhabitat, nest site selection, nesting grounds

## Abstract

The green sea turtle ( *Chelonia mydas* ) is the only sea turtle species that breeds in China, and the largest remaining nesting grounds for green sea turtles in Chinese waters is found on the Qilianyu atoll of the Xisha Islands. Nesting site selection is particularly important for egg survival, and understanding the microhabitat characteristics of green sea turtle nesting sites is crucial for delineating priority conservation areas for nesting grounds. In this study, we aimed to examine the role of several microhabitat ecological factors in the selection of nesting sites and the success of nesting. To this end, we performed differential comparisons, principal component analysis, and generalized linear model analysis. There were significant differences in microhabitat ecological factors, such as surface temperature, humidity, and particle size distribution (0.250–1 mm), between the nesting sites and the surrounding area. Green sea turtle nests were concentrated at a distance of 20.1–30 m from the high tide line, with a preferred distance from vegetation of 0–0.5 m. The vegetation cover of successful nests was concentrated in the range of 0%–25%, and the preferred sand types for successful nests were coarse sand (0.425–1 mm) and medium sand (0.250–0.425 mm). The average hatching success of six green sea turtle nests on North Island was 94.52%. The key microhabitat factors affecting the success of nesting were found to be sand characteristics such as humidity, bulk density, and particle size ratio. Therefore, green sea turtles on the Xisha Islands exhibit preferences for microhabitat ecological factors during nesting site selection, and the ecological characteristics of nesting grounds can affect the hatching success rate of green sea turtles. Therefore, it is recommended to continuously monitor the characteristics of and changes in green sea turtle nesting site selection and take measures to provide high‐quality nesting and hatching environments for sea turtles.

## Graphical abstract

The green sea turtles in the Xisha Islands exhibit nest site microhabitat preference. The nesting sites are mainly concentrated at a distance from vegetation zone of 0–0.5 m, with 92% of aborted nests were distributed under dense vegetation. Green turtles tend to choose areas with coarse sand (0.425–1 mm) and medium sand (0.250–0.425 mm) for nesting. The hatching success rate of green sea turtles is positively correlated with microhabitat characteristics.

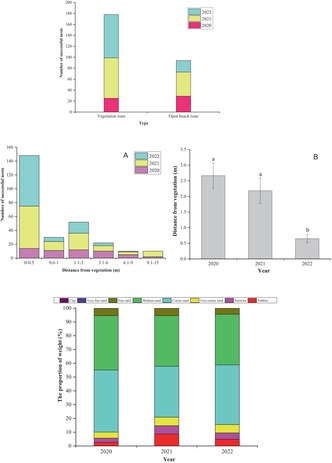

## 1 Introduction

Sea turtles are marine reptiles that spend most of their time in the ocean but return to their natal beaches to lay eggs, and the choice of nesting sites is crucial for egg survival (Bowen and Karl 2007; Lohmann, Putman, and lohmann 2008). Therefore, nest site selection is an important mechanism through which sea turtles adapt to their environment (Kamel and Mrosovsky 2005).

The sea turtle eggs hatching process requires certain levels of temperature, humidity, water potential, salinity, and respiratory gases (Ackerman 1997; López‐Castro, Carmona, and Nichols 2004; Mrosovsky 1994). The microhabitat characteristics of sea turtle nests vary with nest location, vegetation cover, sand characteristics, temperature, and humidity, and these factors can affect the reproductive adaptability of the population (Tezak, Sifuentes‐Romero, and Wyneken 2018; Salleh et al. 2020; Lolavar and Wyneken 2021). The characteristics of sand, such as salinity and grain size, may force females to alter their nesting behavior (Obare et al. 2019), and loose and porous sand is a necessary condition for successful sea turtle nesting and hatching (São Miguel, Anastácio, and Pereira 2022). Wang and Cheng (1999) observed that green sea turtle ( *Chelonia mydas* ) nests were distributed within vegetated areas on Wan‐an Island, Taiwan, suggesting a substantial relationship between vegetation and nest site selection. López‐Castro, Carmona, and Nichols (2004) indicated that humidity and temperature play important roles in the selection of suitable nesting sites by female green sea turtles, as these factors can affect gas exchange and the metabolic processes of embryo development. The elevation of the nest must be sufficiently high to prevent it from being flooded by tides, groundwater, waves, or erosion (Katselidis et al. 2013). However, nests at higher elevations may also experience higher temperatures (Kaska et al. 1998; Zárate et al. 2013; Sari and Kaska 2015; Fadli et al. 2023), and the thermal conditions in the nest affect the development of sea turtle clutches, with high temperatures potentially reducing reproductive success and hatchling quality (Bentley et al. 2020; Rutledge et al. 2024). In addition, the distance from the high tide line is also one of the factors that must be considered (Zavaleta‐Lizárraga and Morales‐Mávil 2013). The high relative humidity (> 1%) of nests located within 10 m of the high tide line can affect embryo development, and the nests will also face the risk of high wave action (Maneja et al. 2021) and tidal erosion (Madden et al. 2008; Veelenturf et al. 2022). Nests closer to inland areas are influenced by factors such as root penetration, arid climates, hatchling disorientation, and predation by natural enemies (Wood and Bjorndal 2000).

The hatching success rates of green sea turtles are largely influenced by the microhabitats surrounding their nests (Noble, Stenhouse, and Schwanz 2018; Stewart, Booth, and Rusli 2019). Research on the selection characteristics of sea turtle nesting sites and microhabitats is vital for the protection of breeding sites and assessment of population health (Kamel and Mrosovsky 2005). The most common research method is the evaluation of the microhabitat characteristics of successful nesting cavities (López‐Castro, Carmona, and Nichols 2004; Turkozan, Yamamoto, and Yilmaz 2011; Zare, Vaghefi, and Kamel 2012; Zavaleta‐Lizárraga and Morales‐Mávil 2013). However, sea turtles sometimes return to the sea without nesting (known as “false crawls”) or dig multiple times before successfully nesting (Weishampel et al. 2003; Nishizawa et al. 2013). The characteristics of aborted nests should be included in the comprehensive evaluation of sea turtle nest site selection, as some studies have suggested that it is more difficult for sea turtles to dig their egg chambers on drier beaches (Salleh et al. 2018).

Qilianyu of the Xisha Islands is currently the largest green sea turtle nesting ground in China (Jia et al. 2019; Wang et al. 2019; Zhang et al. 2024). More than 100 green sea turtle nests have been recorded annually since 2016 (Jia et al. 2019; Zhang et al. 2024). Green sea turtles breeding on the Xisha Islands represent a new geographical population and an independent conservation management unit (Gaillard et al. 2021; Song et al. 2022; Li et al. 2023). Therefore, conservation of and research on green sea turtles on the Xisha Islands are important for maintaining China's sea turtle population.

In this study, we compared the differences in microhabitat ecological factors between usage and control quadrats, and between successful and aborted nests on Qilianyu. The factors affecting hatching success were also identified. We aimed to establish an information database on the reproductive ecology and habitat status of green sea turtles on the Xisha Islands and to provide a scientific reference for the refined management of green sea turtle nesting grounds.

## 2 Materials and Methods

### 2.1 Study Area

**FIGURE 1:**
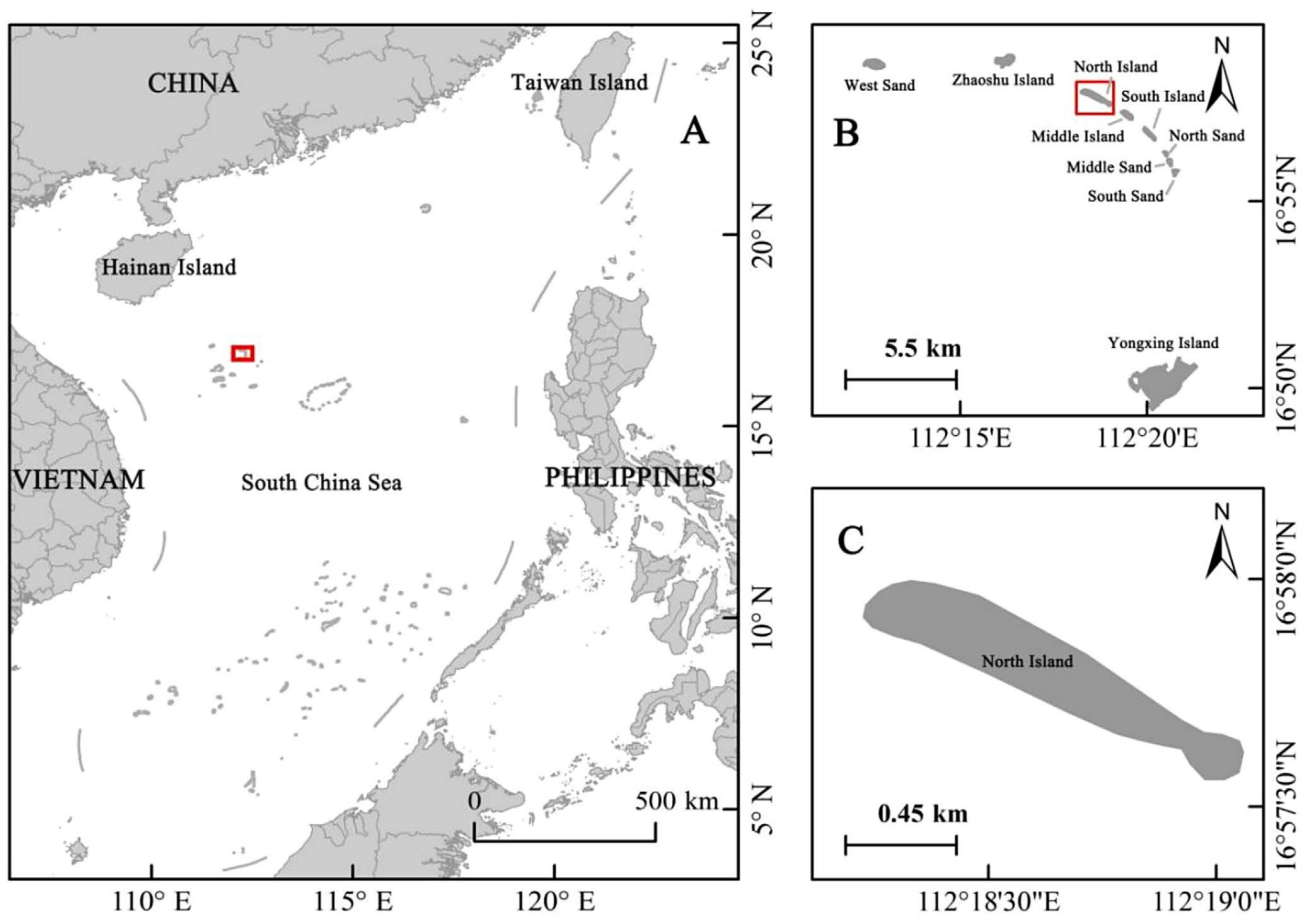
Map of the study area: (A) South China Sea, Xisha Islands are located in the northwestern section of the sea; (B) Qilianyu is a subgroup of islands located in the northeastern Xisha Islands; (C) North Island is located north of Yongxing Island and is part of the Qilianyu atoll of the Xisha Islands.

North Island (16°57.8′ N, 112°18.6′ E) is located north of Yongxing Island and is part of the Qilianyu atoll of the Xisha Islands. The island is long and narrow, running northwest to southeast, with a length of over 1500 m and a width of approximately 290 m. It has an area of about 0.4 km^2^, making it the largest island of the Qilianyu atoll. North Island is heavily vegetated, with the main species including *Scaevola taccada* , *Messerschmidia argentea*, *Pisonia grandis* , *Guettarda speciosa* , and *Terminalia catappa* . From May to September each year, green sea turtles come ashore to lay their eggs, with the number of sea turtle nests on North Island accounting for 63% of those in the Qilianyu region in recent years (Zhang et al. 2024). The island is known as “Sea Turtle Island” (Figure 1).

### 2.2 Monitoring of Nests and Sampling Methods for Microhabitat Ecological Factors

Through nocturnal and subsequent diurnal patrols in 2020–2022, all identified green sea turtle nests were marked with signage (including the laying date and nest number). The coordinates of the nest sites were recorded using a handheld GPS devices (ZL‐188; Zhuolin Electronic Technology Co. Ltd., China).

As female sea turtles may make multiple attempts to dig nests before successfully finding a suitable location for nesting, nests where successful egg laying occurred are referred to as successful nests, whereas aborted holes without eggs are referred to as aborted nests (Olgun et al. 2016).

We designated a 1 × 1 m quadrat centered on a successful nests as the usage quadrat. The chamber of a successful nest was located by investigating the soft sand area behind the covering or “false” body pit left by the female. Randomly selected 1 × 1 m quadrats without nests located every 100 m along North Island were used as control quadrats. The microhabitat ecological factors within the quadrats were measured. Centered on the aborted nests, microhabitat ecological factors within a 1 × 1 m range of aborted nests were measured.

The microhabitat ecological factors of the nests included distance from vegetation, vegetation coverage, distance from the high tide line, sand surface temperature and humidity, and sand characteristics. To measure the ecological factor variables of nests more accurately, these data were collected during low tide (Salleh et al. 2018).

#### 2.2.1 Distance From High Tide Line

Using the location of the nest as a reference point, the distance of the nest from the high tide line was measured using a 30.0 m long measuring tape (± 0.1 m). Following the statistical method of López‐Castro, Carmona, and Nichols (2004), the distance from the high tide line was divided into five intervals: 0–10 m, 10.1–20 m, 20.1–30 m, 30.1–40 m, and > 40 m.

#### 2.2.2 Distance From Vegetation

After the female turtles completed their nests and returned to the sea, the distance of each nest (either successful or aborted) from the vegetation was measured. The distribution type of each nest was classified according to the following two categories: within an open beaches zone (distance of > 1 m from vegetation) or within a vegetation zone (distance of ≤ 1 m from vegetation).

#### 2.2.3 Vegetation Coverage

The percentage of vegetation cover was determined using the vertical projection contour of the vegetation in the quadrat. This was conducted at midday when the shadow of the leaves created contour lines on the sand.

#### 2.2.4 Sand Surface Temperature and Humidity

When the female sea turtles were laying eggs, the surface temperature and relative humidity of the sand in each nest were recorded using a soil thermometer probe (± 1°C) (HKCL‐817; Heistek Technology Co. Ltd., China) inserted at a depth of 5 cm from the surface around the nests.

#### 2.2.5 Sand Characteristics

The sand characteristics of the nests were evaluated by measuring indicators, such as matrix compactness, pore water content, and sand size (Chen, Cheng, and Hong 2007). After the female green sea turtles had finished nesting, three sand samples (350–500 g) were randomly collected from each usage and control quadrat at a depth of 45–60 cm without destroying the nest, as well as from approximately 10 cm outside the aborted nests. The samples were then placed in double‐sealed plastic bags and transported to the laboratory for the analysis of sand characteristics, including pore water content, particle density, bulk density, sand porosity, and proportion of sand particle types.

To determine the pore water content, we measured 200 mL of sand from each sample using a graduated cylinder, which was transferred to a sample bag and weighed using an electronic balance (AL204‐IC; Jin Hua Ke Co. Ltd., China). The samples were dried in an oven at 52°C for at least 24 h and then weighed again (Gardner, 1986). The pore water content (PC) was calculated using the following formula:

$$\mathrm{PC}\;\left(\%\right)=\frac{\text{Preweighed sand}\;\left(g\right)-\text{Dried sand}\;\left(g\right)}{\text{Preweighed sand}\;\left(g\right)}\times 100$$

Particle density, bulk density, and sand porosity indicate the density of solid constituents, mass of material contained within a given volume, and amount of pore space in a sample, respectively (Gravelle and Wyneken 2022). To calculate the particle density, 40 g of dried sand was placed in a 100 mL graduated cylinder containing 50 mL of deionized water. After stirring the replacement air, the volume of the displaced water was recorded. An initial volume of 50 mL was subtracted from this volume, which was equal to the volume of the solid sand. Particle density (gcm^−3^), which is the weight of the dry sand particles divided by the volume of the solid sand, was calculated as follows:

$$\text{Particle density}\;\left(g\cdot {\mathrm{cm}}^{-3}\right)=\frac{\mathrm{Dry}\;\text{sand mass}\;\left(g\right)}{\text{Volume of water displaced}\;\left({\mathrm{cm}}^{3}\right)}$$

The bulk density, which is the mass of sand per unit volume, was measured as the mass of sand required to fill a graduated cylinder to 100 mL (= cm^3^). It was calculated as follows:

$$\text{Bulk density}\;\left(g\cdot {\mathrm{cm}}^{-3}\right)=\frac{\mathrm{Dry}\;\text{mass of}\;100\;{\mathrm{cm}}^{3}\;\text{of sand}\;\left(g\right)}{100\;\left({\mathrm{cm}}^{3}\right)}$$

The sand porosity, defined as the percentage of pore space occupied by sand in relation to the total volume of sand (Tan 1995), was calculated using the following formula:

$$\text{Porosity}\;\left(\%\right)=\frac{\text{Bulk density}\;\left(g\cdot {\mathrm{cm}}^{-3}\right)}{\text{Particle density}\;\left(g\cdot {\mathrm{cm}}^{-3}\right)}\times 100$$

Sand samples were collected from various monitoring points, and dried samples were passed through a standard series of seven sieves: 4.0, 2.0, 1.0, 0.425, 0.250, 0.125, and 0.063 mm (OLOEY, Haichuang Instrument Co. Ltd., China). The samples were then sorted using a vibrating sieve (CHENGJIA, Haichuang Instrument Co. Ltd., China). Materials coarser than 4 Φ (> 63 μm—sand fraction) were analyzed at Φ intervals (−2 to 4 Φ), while samples with particle sizes < 63 μm were subjected to liquid absorption analysis. The sand retained on each sieve and the silt and clay obtained from the pipette analysis were dried again and weighed (± 0.01 g) to determine their proportions in the total mass using the scale and nomenclature of Wentworth (1922) to characterize the sand composition of the nesting beach. The sand grains were classified based on their size as follows: pebbles (> 4 mm), granules (2–4 mm), very coarse sand (1–2 mm), coarse sand (0.425–1 mm), medium sand (0.250–0.425 mm), fine sand (0.125–0.250 mm), very fine sand (0.063–0.125 mm), and clay (< 0.063 mm).

### 2.3 Determination of Hatching Success Rates

The method for determining the hatching success rate of green sea turtles involves counting nest inspections 70 days after the eggs are laid, which is approximately 20 days longer than the mean incubation period (50.8 ± 0.6 days) for this nesting ground (Yao 2021). Nest inspections involve counting the remaining eggshells and intact unhatched eggs in the nest to calculate the total number of eggs in the clutch (Cheng et al. 2008) and the hatching success rate (Staines, Booth, and Limpus 2019). We used the following formula:

$$\begin{array}{c} \text{Hatching success rate}\;\left(\%\right)=\\ \;\frac{\text{The number of total eggs}-\left(\text{Unhatched}+\text{Undeveloped eggs}\right)}{\text{The number of total eggs}}\times 100 \end{array}$$

### 2.4 Data Analysis

Statistical analyses were performed using SPSS version 19.0 and R version 3.6.3. Prior to statistical analysis, all data were tested for normal distribution and homogeneity of variances. For further statistical analysis, different methods were used to test the significance of differences in ecological factors between the usage and control quadrats, with normal distribution and homogeneity of variances assessed using *t*‐tests, while non‐parametric Mann–Whitney *U*‐tests were used for other ecological factors. To further analyze the characteristics of green sea turtle nest site selection, principal component analysis was conducted on the ecological factors of the nesting sites to determine the main factors influencing nest site selection. One‐way analysis of variance (ANOVA) was used to determine the differences in ecological factor selection between successful and aborted nests, and a generalized linear model (GLM) was used to extract key microhabitat factors that determine successful nesting. Successful nests were treated as the response variables (binomial distribution). Distance from the high tide line, distance from vegetation, vegetation coverage, humidity, temperature, bulk density, and particle size ratio (0.250–1 mm) were included as predictor variables. First, a full model was built, and the predictor variables with collinearity were removed based on the coefficient of the variance inflation factor (VIF) (VIF > 5 was considered to indicate collinearity). Finally, a stepwise regression model selection was performed based on the Akaike Information Criterion (AIC) value, and the final model was subjected to Pearson's chi‐squared test. The significance of the predictor variables in the selected model was tested using a likelihood ratio test. Pearson's correlation analysis was used to assess the effects of ecological factors on the hatching success of green sea turtles (Chen, Cheng, and Hong 2007; Pike 2008). The relevant data in the study were presented as mean ± standard deviation (Mean ± SD), with *p* < 0.05 indicating significant differences and *p* < 0.01 indicating extremely significant differences (two‐tailed test) (Zare, Vaghefi, and Kamel 2012).

## 3 Results

### 3.1 Microhabitat Ecological Factors in the Usage and Control Quadrats

We conducted a significance test on the differences in microhabitat ecological factors between usage quadrats (*n* = 38) and control quadrats (*n* = 38) at green sea turtle nesting sites and found that among the nine ecological factors, surface temperature (*p* = 0.017) exhibited a significant difference, whereas humidity (*p* = 0.001) and the particle size ratio (0.250–1 mm) (*p* = 0.001) showed extremely significant differences (Table 1).

**TABLE 1:** Evaluated ecological factors for the usage and control quadrats and their determined values.

Ecological factors	Mean ± SD		*p*
	Usage quadrat (*n* = 38)	Control quadrat (*n* = 38)	
Distance from the high tide line (m)	13.14 ± 6.23	11.77 ± 4.52	0.275
Distance from vegetation (m)	3.01 ± 3.20	2.25 ± 1.97	0.214
Vegetation coverage (%)	23.42 ± 36.26	11.32 ± 23.27	0.087
Surface temperature (°C)	30.26 ± 7.37	33.39 ± 2.57	0.017*
Humidity (%)	5.13 ± 1.75	3.76 ± 0.93	0.001**
Bulk density (gcm^−3^)	1.38 ± 0.06	1.42 ± 0.12	0.148
Particle density (gcm^−3^)	2.35 ± 0.09	2.44 ± 0.19	0.131
Sand porosity (%)	39.86 ± 2.75	44.76 ± 10.88	0.087
Particle size (0.250–1 mm) ratio (%)	84.39 ± 8.04	55.37 ± 25.27	0.001**

* *p* < 0.05, significant difference.

** *p* < 0.01, highly significant difference.

Eight ecological factors in the usage quadrats were selected for principal component analysis. The analysis results showed that the cumulative contribution rate of the first three principal components with eigenvalues > 1 was 72.635%. The eigenvalues of the three principal components were 3.407, 1.367, and 1.037. For the first principal component, the absolute values of the loadings of distance from vegetation, vegetation cover, and sand bulk density were relatively large. For the second principal component, the absolute values of the distance from the high tide line and the particle size ratio (0.250–1 mm) were relatively large. For the third principal component, the absolute value of the loading of humidity was relatively large.

These results indicate that microhabitat ecological factors such as sand bulk density, distance from vegetation, vegetation cover, sand particle size, distance from the high tide line, and humidity affected the nest site selection of green sea turtles (Table 2).

**TABLE 2:** Loading of ecological factors.

Ecological factors	Principal components		
	1	2	3
Distance from the high tide line	0.560	0.601	−0.192
Distance from vegetation	0.762	0.270	−0.187
Vegetation coverage	−0.734	−0.072	0.498
Temperature	−0.452	0.515	0.382
Humidity	0.618	0.322	0.583
Bulk density	0.906	−0.18	0.101
Porosity	−0.664	0.231	−0.462
Particle size ratio (0.250–1 mm)	−0.356	0.688	−0.084
Variance explained (%)	42.582	17.086	12.967
Cumulative proportion of variance explained (%)	42.582	59.668	72.635

### 3.2 Microhabitat Ecological Factors in Successful and Aborted Nests

#### 3.2.1 Distance From High Tide Line

A total of 165 records of the distance of successful nests from the high tide line were recorded on North Island of Qilianyu from 2020 to 2022 (Table 3). The majority of successful nests were located within a distance of 0–40 m from the high tide line, accounting for a high proportion of 98.79%. Successful nests located at distances greater than 40 m only accounted for 1.21% of the total recorded successful nests. The highest number of successful nests was found at a distance of 20.1–30 m from the high tide line, accounting for 44.24% of the total, followed by those at a distance of 10.1–20 m (29.09%). The distance of successful nests from the high tide line was significantly greater than that of aborted nests from 2020 to 2022 (Table 4). Furthermore, an analysis of the distance of successful nests from the high tide line for each year from 2020 to 2022 revealed a significant increase in the average distance (*F* = 78.64, *p* = 0.001) (Table 4).

**TABLE 3:** Distribution of successful nests based on distance from the high tide line.

Year	Number of nests (%)					Total
	0–10 m	10.1–20 m	20.1–30 m	30.1–40 m	> 40 m	
2020	14 (32.56)	22 (51.16)	6 (13.95)	1 (2.33)	0 (0.00)	43
2021	1 (2.38)	18 (42.86)	23 (54.76)	0 (0.00)	1 (2.38)	42
2022	0 (0.00)	8 (10.00)	44 (55.00)	27 (33.75)	1 (1.25)	80
Total	15 (9.09)	48 (29.09)	73 (44.24)	28 (16.97)	2 (1.21)	165

**TABLE 4:** Distances of successful and aborted nests from high tide line in different years.

Year	Successful nests	Aborted nests	*p*
2020	13.47 ± 6.59^a^ (*n* = 42)	11.70 ± 4.21 (*n* = 43)	0.008
2021	21.31 ± 5.32^b^ (*n* = 43)	22.83 ± 5.59 (*n* = 30)	0.383
2022	27.46 ± 5.60^c^ (*n* = 80)	26.95 ± 3.61 (*n* = 75)	0.004
Significant result	*F* = 78.64, *p* = 0.001		

*Note:* Different lowercase letters indicate significant differences between different years.

The analysis of beach surface temperature and humidity around nests with different gradients from the high tide line revealed that as the distance of successful nests from the high tide line increased, the temperature increased, whereas the humidity decreased (Table 5).

**TABLE 5:** Surface temperature and humidity of successful nests at different distances from the high tide line.

Factors	Year	0–10 m	10.1–20 m	20.1–30 m	30.1–40 m
Surface temperature (°C)	2020	31.79 ± 0.82	31.82 ± 0.84	31.88 ± 0.75	32.30 ± 0.00
	2021	−	−	−	−
	2022	−	−	30.87 ± 0.55	30.95 ± 0.51
Humidity (%)	2020	5.63 ± 0.88	5.55 ± 1.24	5.25 ± 1.24	4.20 ± 0.00
	2021	−	−	−	−
	2022	−	−	−	−

*Note:* “−” indicates no data.

#### 3.2.2 Distance From Vegetation and Vegetation Coverage

**FIGURE 2:**
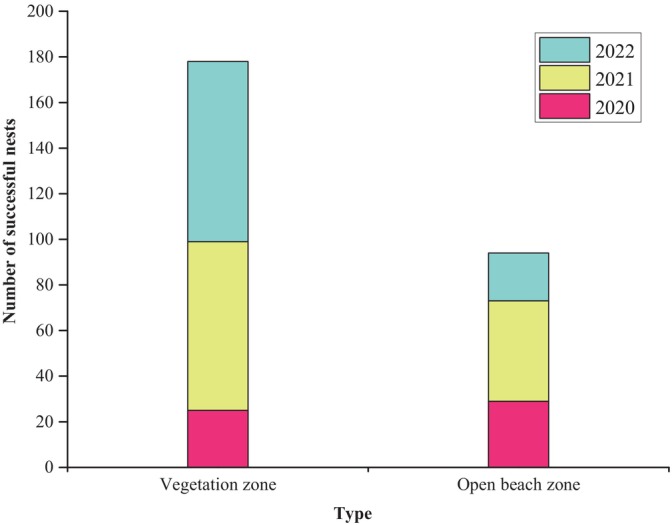
Habitat distribution of successful nests from 2020 to 2022.

From 2020 to 2022, 272 measurements of the distance of successful nests from the vegetation zone were taken, which were divided into two distribution types: within the vegetation zone (distance to vegetation < 1 m) and in the open beach zone (distance to vegetation > 1 m). The results showed that green sea turtles tended to choose areas close to the vegetation zone for nesting, with approximately 65.44% (178 nests) of successful nests distributed within the vegetation zone. The number of nests distributed in the vegetation zone increased year by year, with 46.30% in 2020, 62.71% in 2021, and 79% in 2022 (Figure 2). The average vegetation coverage of all green sea turtle nests was 28.64% (*n* = 272), with approximately 76.47% of the nests distributed in areas with vegetation coverage of 0%–50%, and only 8.46% of nests distributed in areas with vegetation coverage of 51%–75% (Table 6).

**TABLE 6:** Distribution of successful nests based on vegetation coverage.

Year	Number of nests (%)				Total
	0%–25%	26%–50%	51%–75%	76%–100%	
2020	36 (66.67)	7 (12.96)	4 (7.41)	7 (12.96)	54
2021	62 (52.54)	19 (16.10)	10 (8.47)	27 (22.88)	118
2022	62 (62.00)	22 (22.00)	9 (9.00)	7 (7.00)	100
Total	160 (58.82)	48 (17.65)	23 (8.46)	41 (15.07)	272

**FIGURE 3:**
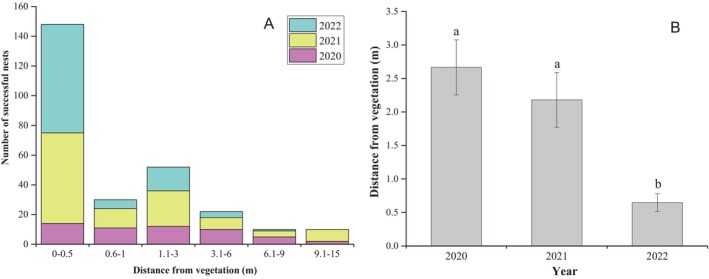
(A) Distribution of successful nests based on distance from vegetation; (B) annual change in distance of successful nests from vegetation from 2020 to 2022. The “a” and “b” labels above the error bars in Panel B indicate significant differences at the *p* < 0.05 level.

Therefore, green sea turtles prefer nesting sites closer to the vegetation zone, with a preferred distance of 0–0.5 m (148 nests, accounting for 54.41% of the total), as shown in Figure 3A. From 2020 to 2022, the average distance from vegetation decreased annually, with the distance in 2022 being significantly lower than that in 2020 and 2021 (*p* = 0.001), as shown in Figure 3B.

In 2022, a total of 75 excavation failures (aborted nests) by green sea turtles were recorded on North Island of Qilianyu (Table 7), with 69 attempts in the vegetation zone and 6 attempts in the open beach zone, indicating that 92% of the aborted nests were distributed in the vegetation zone.

**TABLE 7:** Distribution types of aborted nests.

Year	Number of nests (%)		Total
	Vegetation zone	Open beach area	
2020	16 (35.56)	29 (64.44)	45
2021	21 (67.74)	10 (32.26)	31
2022	69 (92.00)	6 (8.00)	75
Total	106 (70.20)	45 (29.80)	151

#### 3.2.3 Sand Characteristics

The pore water content, particle density, bulk density, and sand porosity of the successful and aborted nests were analyzed. The results showed that there were no significant differences in the parameters of particle density (*p* = 0.739), bulk density (*p* = 0.229), and sand porosity (*p* = 0.735) of successful nests during the study years. The bulk density of aborted nests increased annually from 2020 to 2022, and the bulk density in 2022 was significantly higher than that in 2020 and 2021 (*p* = 0.043). The particle density and sand porosity of aborted nests decreased annually, and both the particle density (*p* = 0.007) and sand porosity (*p* = 0.002) were significantly higher in 2021 and 2022 than in 2020 (Table 8).

**TABLE 8:** Microhabitat ecological factors in successful and aborted nests from 2020 to 2022.

Year	Pore water (%)		Bulk density (gcm^−3^)		Particle density (gcm^−3^)		Porosity (%)	
	Successful nests	Aborted nests	Successful nests	Aborted nests	Successful nests	Aborted nests	Successful nests	Aborted nests
2020	—	—	1.42 ± 0.06^a^ (*n* = 17)	1.33 ± 1.96^Ab^ (*n* = 10)	2.35 ± 0.09^a^ (*n* = 17)	2.44 ± 1.84^Ab^ (*n* = 10)	39.86 ± 2.75^a^ (*n* = 17)	44.76 ± 10.88^Ab^ (*n* = 10)
2021	—	—	1.41 ± 1.82^a^ (*n* = 30)	1.39 ± 0.11^Aa^ (*n* = 16)	2.36 ± 0.13^a^ (*n* = 30)	2.35 ± 0.13^Ba^ (*n* = 16)	40.12 ± 5.00^a^ (*n* = 30)	40.70 ± 5.39^Ba^ (*n* = 16)
2022	2.46 ± 1.82^a^ (*n* = 42)	4.42 ± 1.43^b^ (*n* = 45)	1.40 ± 0.07^a^ (*n* = 42)	1.42 ± 0.07^Ba^ (*n* = 45)	2.32 ± 0.13^a^ (*n* = 42)	2.28 ± 0.13^Ba^ (*n* = 45)	39.31 ± 4.49^a^ (*n* = 42)	37.47 ± 4.63^Ba^ (*n* = 45)

*Note:* Different lowercase letters indicate significant differences between successful and aborted nests for each ecological factor; different uppercase letters indicate significant differences in the ecological factors of aborted nests in different years; “‐” indicates not detected.

In 2022, the pore water content in the sand surrounding successful nests was significantly lower than that in the sand surrounding aborted nests (*p* = 0.031). In 2020, the bulk density (*p* = 0.001) of the sand in successful nests was significantly higher, but the particle density (*p* = 0.02) and sand porosity (*p* = 0.001) were significantly lower than those in aborted nests (Table 8).

**FIGURE 4:**
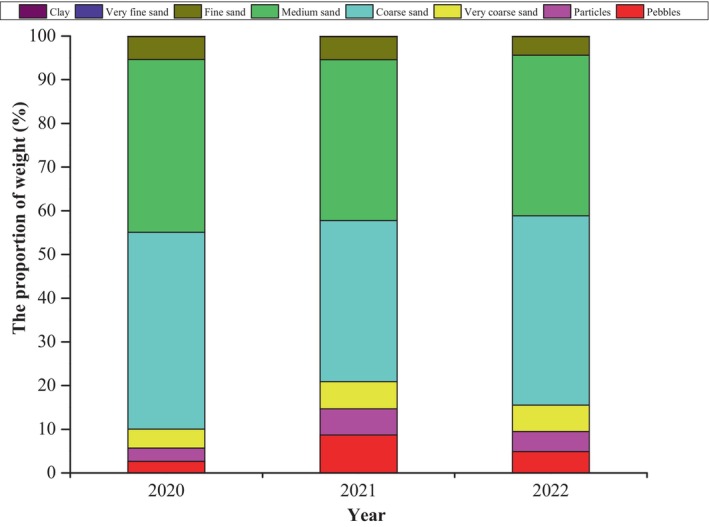
Sand grain types of successful nests.

From 2020 to 2022, the sand grain types collected from the successful nests ranged from coarse pebbles (> 4 mm) to very fine sand (0.063–0.125 mm) and clay particles (0.004–0.063 mm). The distribution of these sand grain types, ranked by average weight percentage, was as follows: coarse sand (41.77%), medium sand (37.70%), very coarse sand (5.52%), pebbles (5.42%), fine sand (4.91%), particles (4.54%), very fine sand (0.08%), and clay (0.06%) (Figure 4). There was no significant difference in the weight percentage of sand grain types over the 3 years. Although there were significant differences in the distribution of sand grain types among the different successful nests, most sand types in the successful nests were coarse sand (0.425–1 mm) and medium sand (0.250–0.425 mm).

**FIGURE 5:**
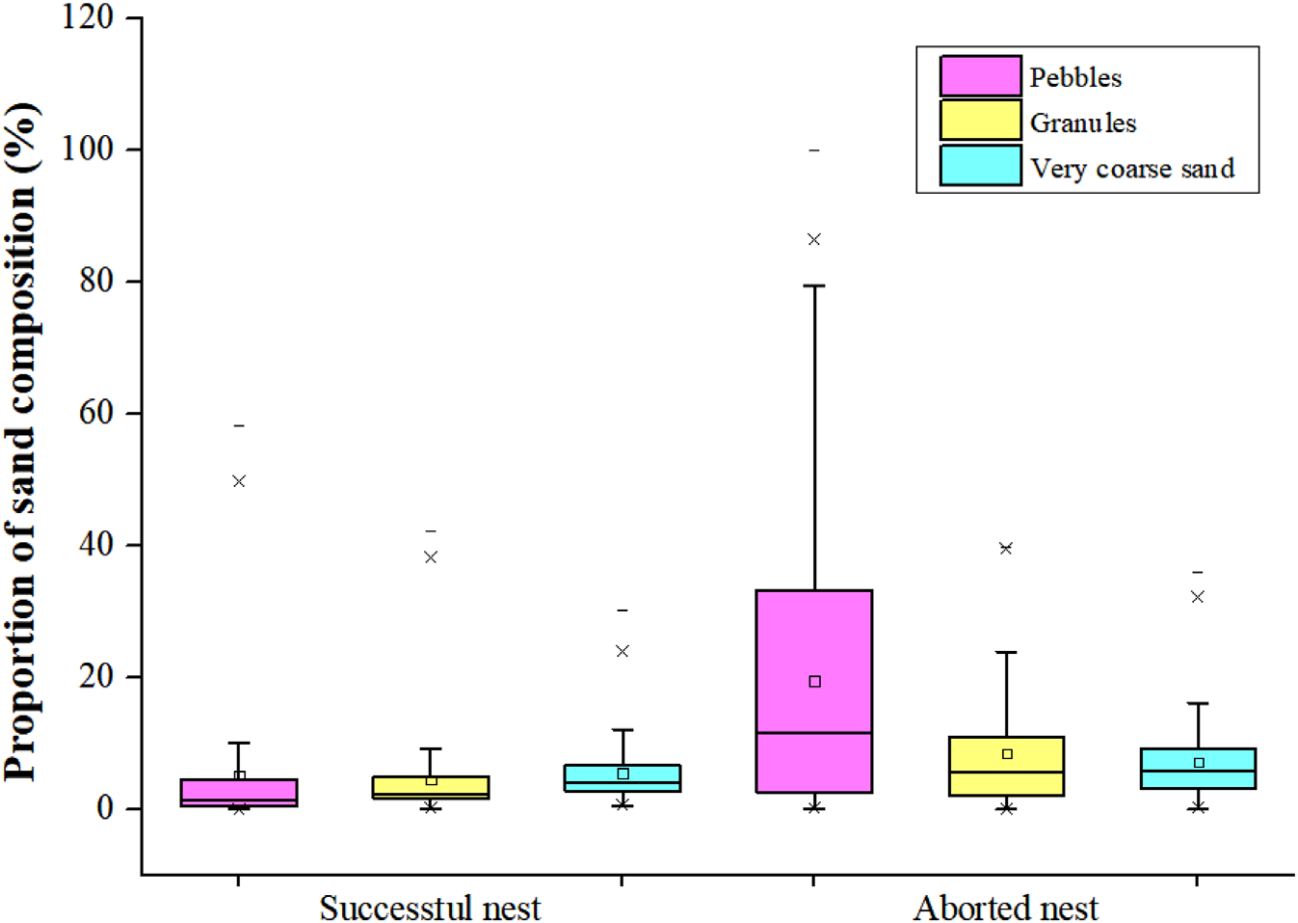
Sand types distribution in successful and aborted nests from 2020 to 2022. The solid horizontal lines from the top to the bottom of each box plot indicate the maximum value, 75% quartile, median, 25% quartile, and minimum value. Empty boxes indicate average values.

The sand type distributions in successful and aborted nests from 2020 to 2022 were compared. The proportions of pebbles, granules, and very coarse sand in sand samples from aborted nests were higher than those in samples from successful nests, with mean percentage differences of 14.42 (*p* = 0.001), 4.02 (*p* = 0.001), and 1.70 (*p* = 0.022), respectively (Figure 5).

#### 3.2.4 Key Microhabitat Factors Affecting the Success of Nesting

Full datasets were obtained for 39 successful and 40 aborted nests (79 in total), and GLM analyses were applied to these datasets. Variables with multiple collinearities were excluded, and stepwise model selection in the GLM analysis resulted in a logistic regression model containing only humidity, bulk density, and the particle size ratio (0.250–1 mm) as explanatory variables. The effects of humidity (*Z* = 3.240, *p* = 0.001), bulk density (*Z* = −2.970, *p* = 0.003), and the particle size ratio (*Z* = 3.683, *p* = 0.0002) on nesting success were extremely significant (Table 9).

**TABLE 9:** Results of generalized linear model predicting the key microhabitat factors determining successful nesting.

Response variable	Predictor variable	Estimate	SE	*Z*	2.5%	97.5%	*p*
Successful nests	Humidity	1.597	0.493	3.240	0.772	2.759	0.001**
	Bulk density	−19.219	6.472	−2.970	−33.629	−6.629	0.003**
	Particle size ratio	0.134	0.036	3.683	0.074	0.220	0.0002***

** An extremely significant correlation at the 0.01 level (bilateral).

*** An extremely significant correlation at the 0.001 level (bilateral).

### 3.3 Effects of Microhabitat Ecological Factors in Nests on the Hatching Success

In 2022, six green sea turtle nests were monitored for hatching success and surrounding microhabitat characteristics. The average hatching success rate of these six nests was 94.52%. The characteristics of microhabitat ecological factors within the nests are shown in Table 10. The correlation analysis results indicated that the hatching success rate was positively correlated with microhabitat characteristics such as soil pore water content (*r* = 0.513, *n* = 6, *p* = 0.298), particle density (*r* = 0.387, *n* = 6, *p* = 0.449), porosity (*r* = 0.514, *n* = 6, *p* = 0.297), humidity (*r* = 0.453, *n* = 6, *p* = 0.367), and the particle size ratio (0.250–1 mm) (*r* = 0.397, *n* = 6, *p* = 0.435) around the nests. The hatching success rate was negatively correlated with soil bulk density (*r* = −0.418, *n* = 6, *p* = 0.410). However, the hatching success rate had little correlation with distance from the high tide line (*r* = −0.241), distance from vegetation cover (*r* = 0.103), and sand surface temperature (*r* = 0.259) (Table 11).

**TABLE 10:** Hatching success of nests and the surrounding microhabitat ecological factors.

Nest number	Hatching success (%)	Pore water (%)	Bulk density (gcm^−3^)	Particle density (gcm^−3^)	Porosity (%)	Particle size ratio (0.250–1 mm) (%)	Distance from the high tide line (m)	Distance from vegetation (m)	Vegetation coverage (%)	Surface temperature (°C)	Humidity (%)
48	95.68	4.23	1.422	2.11	32.46	83.96	32.5	0	20	31	22
19	96.67	4.61	1.353	2.11	35.73	88.85	26	0	15	32	51
35	96.77	3.04	1.44	2.35	38.80	85.69	28.7	0	10	31	25
51	86.27	1.41	1.433	2.11	31.93	84.97	30.8	0	10	31	12
65	95.37	1.07	1.402	2.11	33.41	85.89	26	2.5	0	31	73
66	96.33	2.30	1.333	2.35	43.35	89.36	32.3	0	30	31	29
Mean ± SD	94.52 ± 4.08	2.78 ± 1.45	1.40 ± 0.04	2.19 ± 0.12	35.95 ± 4.42	86.45 ± 2.17	29.38 ± 2.95	0.42 ± 1.02	14.17 ± 10.21	31.17 ± 0.41	35.33 ± 22.51

**TABLE 11:** Correlation between ecological factors and hatching success.

Factors	Pore water	Bulk density	Particle density	Porosity	Particle size ratio (0.250**–**1 mm)	Distance from the high tide line	Distance from vegetation	Vegetation coverage	Surface temperature	Humidity	Hatching success
Pore water	1										
Bulk density	−0.187	1									
Particle density	−0.057	−0.186	1								
Porosity	0.049	−0.598	0.899*	1							
Particle size ratio (0.250–1 mm)	0.138	−0.921**	0.383	0.728	1						
Distance from the high tide line	0.028	0.068	0.293	0.19	−0.221	1					
Distance from vegetation	−0.576	0.053	−0.316	−0.281	−0.127	−0.561	1				
Vegetation coverage	0.027	−0.729	0.637	0.841*	0.833*	0.268	−0.476	1			
Temperature	0.618	−0.488	−0.316	−0.024	0.541	−0.561	−0.2	0.183	1		
Humidity	−0.139	−0.343	−0.287	−0.071	0.302	−0.802	0.820*	−0.233	0.341	1	
Hatching success	0.513	−0.418	0.387	0.514	0.397	−0.241	0.103	0.118	0.259	0.453	1

* An extremely significant correlation at the 0.05 level (bilateral).

** An extremely significant correlation at the 0.01 level (bilateral).

There were also certain correlations among ecological factors: sand humidity was significantly positively correlated with the distance of successful nests from vegetation (*r* = 0.820, *n* = 6, *p* = 0.046), porosity was significantly positively correlated with particle density (*r* = 0.899, *n* = 6, *p* = 0.015), and vegetation coverage was positively correlated with porosity (*r* = 0.841, *n* = 6, *p* = 0.036) and the particle size ratio (0.250–1 mm) (*r* = 0.833, *n* = 6, *p* = 0.039). The particle size ratio (0.250–1 mm) was negatively correlated with bulk density (*r* = −0.921, *n* = 6, *p* = 0.009) (Table 11).

Therefore, microhabitat ecological factors in the nests can influence the hatching success of green sea turtles. Within a certain range, higher sand pore water content, particle density, porosity, humidity, proportion of particle size (0.250–1 mm), and lower sand bulk density were associated with higher hatching success rates of green sea turtles.

## 4 Discussion

The characteristics of nest site selection by sea turtles can affect their reproductive fitness and the survival of populations (Whitemore and Dutton 1985). Our research indicates that green sea turtles on the Xisha Islands exhibit selectivity toward certain microhabitat ecological factors at nesting sites. The key microhabitat factors affecting the success of nesting were sand characteristics such as humidity, bulk density, and particle size ratio. The ecological factors of nesting grounds can affect the hatching success of green sea turtles.

### 4.1 The Influence of Distance From High Tide Line on Nest Site Selection

On North Island of Qilianyu, the optimal nesting site selection for green sea turtles was at a distance of 20.1–30 m from the high tide line, with nests located more than 40 m away accounting for only 1.21% of all statistically recorded nests. Our findings are consistent with the conclusions of López‐Castro, Carmona, and Nichols (2004), who found a higher nesting success rate between 20 and 30 m from the high tide line. They suggested that nests located more than 40 m away had drier and more compact sand with lower relative humidity, which made it difficult for the sea turtles to nest. The average beach width on North Island is 21.3 m, but with only a few areas having beaches that provide a distance of 20–30 m from the high tide line. To build a safer nests, some sea turtles crawl into dense vegetation to dig nests under bushes. Furthermore, Stokes, Esteban, and Hays (2024) found that sea turtles tend to crawl a sufficient distance to minimize the seawater overwash of nests, which can kill embryos.

Our results showed that, as the distance of the nest from the high tide line increased, the temperature increased and humidity decreased, similar to the findings of Sarahaizad, Shahrul Anuar, and Mansor (2012). The optimal relative humidity around the nesting site for green sea turtles was < 1%, with an optimal temperature range of 27°C–32°C (Obare et al. 2018). However, in 2020, the surface humidity and temperature of the beach on North Island were generally higher, with a humidity range of 3.7%–7.6% and a temperature range of 30.3°C–33.2°C. However, the abrupt temperature increase reported at nest sites by Stoneburner and Richardson (1981) may have been caused by warmer sand being brought to the surface when the turtles started to dig. Booth and Freeman (2006) compared sand temperatures at various depths and suggested that nest temperatures differed greatly from surface sand temperatures. The temperature that results in the production of an equal sex ratio in hatchlings is called the pivotal temperature (Kaska et al. 1998). Incubation above the pivotal temperature results in more females, and incubation below the pivotal temperature results in more males. Therefore, variations in beach temperature can cause differences in sex ratios among populations (Sari and Kaska 2015). Slight changes in temperature within the transitional range of temperatures at which both sexes are produced can affect embryo development, the sex ratio, and hatchling quality (Bentley et al. 2020; Rutledge et al. 2024). Therefore, future investigations should include the recording of nest temperatures.

### 4.2 The Influence of Vegetation on Nest Site Selection

The presence of coastal vegetation is a characteristic of nesting beaches, and it helps maintain the stability of temperature and humidity within sea turtle nests (Wood and Bjorndal 2000; Turkozan, Yamamoto, and Yilmaz 2011; Anshary, Setyawati, and Yanti 2014). Vegetation camouflages nests and regulates the temperature of the sand through shading and slight compaction (Horrocks and Scott 1991). Moderate vegetation coverage (10%–30%) contributes to sand accumulation and nest stability, but low vegetation coverage (< 10%) can lead to nest cavity collapse, whereas extremely high vegetation coverage (> 40%) can affect the digging success of green sea turtles because of increased surface density associated with roots (Chen, Cheng, and Hong 2007). In our study, 65.44% of successful nests on North Island were distributed within 1 m of the vegetation edge, and the preferred distance was 0–0.5 m. The average vegetation coverage of all successful nests was 28.64%, which was within the suitable range.

The presence of short root systems in vegetation zones may contribute to the construction of nesting sites; however, large shrub roots may interfere with this process (Mortimer 1990). Wang and Cheng (1999) proposed that green sea turtles may encounter obstacles during their excavation attempts owing to the penetration of tree roots into the sand. Our study confirms this conclusion, as 92% of aborted nests were distributed under dense vegetation. This may be related to the dense roots of *Scaevola taccada* in the vegetation zone, which hindered the turtles from excavating their nests.

Furthermore, vegetation and roots may affect the hatching and emergence success of sea turtles (Redding, Castorani, and Lasala 2024). Staines, Booth, and Limpus (2019) found that loggerhead clutches on Mon Repos beach, Australia that were relocated into shaded nest sites and surrounded by ground vegetation had poorer hatching and emergence success (73% and 66%, respectively) than clutches that were relocated to sun‐exposed nest sites without ground vegetation (79% and 83%, respectively). The authors attributed this to the presence of grass roots penetrating the nest, absorbing moisture, and suffocating the incubated eggs. Similarly, Redding, Castorani, and Lasala (2024) recently published findings from a long‐term dataset (1987–2022) on loggerhead sea turtles nesting across Casey Key, Florida. They showed that root presence decreased hatching success by 21% and emergence success by 18% compared with that of nests without roots. Therefore, future studies should focus on the effects of vegetation roots on sea turtle hatching success.

### 4.3 The Influence of Sand Characteristics on Nest Site Selection

Sand is composed of solid particles, water, and air, which are crucial for embryo development and hatchling survival (Mortimer 1990). The texture of sand determines soil cohesion, water‐holding capacity, structure, and nutrient retention (Gachene and Kimaru 2003).

Green sea turtles nest in locations with different sand particle sizes (Chen, Cheng, and Hong 2007). Mortimer (1990) found that the number of nests was correlated with the average diameter of sand particles and that sea turtles had difficulty constructing suitable nests in rough and dry sand. Our results indicate that the majority of nest cavities contained coarse sand (0.425–1 mm) and medium sand (0.250–0.425 mm), accounting for 79.47% of the total, whereas more pebbles, particles, and very coarse sand (particle size > 1 mm) were found in aborted nests, consistent with the findings of Mortimer (1990). Thus, our findings suggest that green sea turtles prefer areas with sand particle sizes ranging from 0.425 to 1 mm for nesting.

The sand grain size in green turtle nests may influence the nest microenvironment and hatchling performance (Stewart, Booth, and Rusli 2019). Our study showed that the hatching success rate was positively correlated with the particle size ratio (0.250–1 mm). Larger sand particles provide greater porosity, resulting in better ventilation and lower salt content in the nest, because salt can be more effectively washed away by rainwater (Foley, Peck, and Harman 2006). Therefore, larger sand particles have a positive effect on hatching success (Booth, Staines, and Reina 2022). Although sand with a larger particle size holds less water, frequent rainfall events during incubation ensured that embryos remained well hydrated; therefore, a lack of water was unlikely to adversely influence embryonic development (Stewart, Booth, and Rusli 2019). However, Saito et al. (2019) believed that in terms of the survival rate, the coarse sand nest group had significantly lower hatching and emergence success rates and a higher possibility of pip deaths due to the fact that their cavities are easier to collapse. Therefore, further mores studies on the effects of variations in sand particle size and distribution on the incubation of sea turtle eggs are needed.

### 4.4 Implications for Conservation

Xisha Islands are important nesting grounds for green sea turtles; thus, steps should be taken to protect this habitat. The results of our study establish a baseline dataset for developing effective conservation strategies and management plans. Based on our results demonstrating the influence of vegetation and distance from the high tide line in nesting selection and success, interventions such as segmentation and stratification should be considered to clear the aboveground and belowground parts of the vegetation community without compromising its ability to prevent wind erosion and sand fixation. This will create more suitable areas further from the high tide line for green sea turtle nesting. Additional research is required to further elucidate the effect of ecological factors, such as vegetation roots and sand particle size, on sea turtle hatching success.

## 5 Conclusion

Green sea turtles nesting on the Xisha Islands exhibit nest site microhabitat preferences. The nesting sites were mainly concentrated at a distance of 20.1–30 m from the high tide line, with a preferred distance from vegetation of 0–0.5 m. The vegetation cover of nesting sites was primarily between 0% and 25%, and female turtles tended to choose areas with coarse sand (0.425–1 mm) and medium sand (0.250–0.425 mm) for nesting. In addition, the hatching success rate was likely to increase slightly with increasing soil pore water content, particle density, porosity, humidity, and particle size ratio (0.250–1 mm) around the nests, but it decreased slightly with increasing soil bulk density. In conclusion, green sea turtles nesting on the Xisha Islands select nest sites with certain characteristics, which affect the hatching success rate. Fortunately, we observed high hatching success in green sea turtles on the Xisha Islands. The results of this study serve as a valuable reference for sea turtle habitat conservation measures.

## Author Contributions

**Ting Zhang:** conceptualization (equal), formal analysis (equal), investigation (equal), methodology (equal), writing – original draft (equal). **Xiaoyu An:** investigation (equal), writing – review and editing (equal). **Chenglong Zhang:** formal analysis (equal), writing – review and editing (equal). **Yunteng Liu:** formal analysis (equal), writing – review and editing (equal). **Yupei Li:** writing – review and editing (equal). **Yangfei Yu:** investigation (equal), methodology (equal). **Jichao Wang:** conceptualization (equal), formal analysis (equal). **Liu Lin:** conceptualization (equal), writing – review and editing (equal). **Hai‐Tao Shi:** conceptualization (equal), writing – review and editing (equal).

## Conflicts of Interest

The authors declare no conflicts of interest.

## Data Availability

Data are available from the Dryad Digital Repository: https://datadryad.org/stash/share/4aAFQDYLHWIJuJBnpvIhLzow5CjUs2_8zCL2DLXTlg4.
